# The proinflammatory cytokines IL-2, IL-15 and IL-21 modulate the repertoire of mature human natural killer cell receptors

**DOI:** 10.1186/ar2336

**Published:** 2007-12-03

**Authors:** Casimir de Rham, Sylvie Ferrari-Lacraz, Sabrina Jendly, Gregory Schneiter, Jean-Michel Dayer, Jean Villard

**Affiliations:** 1 Transplantation Immunology Unit, Department of Internal Medicine, University Hospital, rue Micheli-du-Crest, Geneva 14, 1211, Switzerland; 2 Division of Immunology and Allergy, Department of Internal Medicine, University Hospital, rue Micheli-du-Crest, Geneva 14, 1211, Switzerland

## Abstract

Natural killer (NK) cells play a crucial role in the immune response to micro-organisms and tumours. Recent evidence suggests that NK cells also regulate the adaptive T-cell response and that it might be possible to exploit this ability to eliminate autoreactive T cells in autoimmune disease and alloreactive T cells in transplantation. Mature NK cells consist of a highly diverse population of cells that expresses different receptors to facilitate recognition of diseased cells and possibly pathogens themselves. *Ex vivo *culture of NK cells with cytokines such as IL-2 and IL-15 is an approach that permits significant expansion of the NK cell subpopulations, which are likely to have potent antitumour, antiviral, or immunomodulatory effects in autoimmunity. Our data indicate that the addition of IL-21 has a synergistic effect by increasing the numbers of NK cells on a large scale. IL-2 and IL-15 may induce the expression of killer cell immunoglobulin-like receptors (KIRs) in KIR-negative populations, the c-lectin receptor NKG2D and the natural cytotoxic receptor NKp44. The addition of IL-21 to IL-15 or IL-2 can modify the pattern of the KIR receptors and inhibit NKp44 expression by reducing the expression of the adaptor DAP-12. IL-21 also preserved the production of interferon-γ and enhanced the cytotoxic properties of NK cells. Our findings indicate that the proinflammatory cytokines IL-2, IL-15 and IL-21 can modify the peripheral repertoire of NK cells. These properties may be used to endow subpopulations of NK cells with specific phenotypes, which may be used in *ex vivo *cellular immunotherapy strategies.

## Introduction

Natural killer (NK) cells are an important population of lymphocytes that originally were regarded to play crucial roles in protection from infectious disease and destroying tumour cells; they are also involved in certain autoimmune diseases and in rejection of transplanted tissues [1,2]. NK cells express many different germline encoded activating or inhibitory receptors that do not rearrange, in contrast to T and B cells, which might suggest that NK cells are unable to respond to more than a limited number of stimuli [3]. The human NK receptors are characterized by genetic diversity, and NK cells were found to express only a subset of these receptors [4,5]. NK cells can be divided into CD56^bright ^and CD56^dim ^subpopulations, the former being more inclined to produce cytokines such as IFN-γ and the latter to lyse target cells [6]. Several activating and inhibitory NK cell receptors have been well characterized, of which killer cell immunoglobulin-like receptors (KIRs), c-type lectins, and natural cytotoxicity receptors (NCRs). Although inhibitory receptors neutralize NK cells, activating receptors are responsible for NK cell activity [3,7-9]. The NK repertoire and its modulation at the cell surface is incompletely understood. Many of the activating receptors are constitutively expressed on all NK cells, and it is actually the increased expression of their ligands on other cells induced by mild stimuli that underpins the diversity of activating receptors.

The genetic diversity of NK cell receptors and the range of diseases in which they are thought to play specific roles would suggest that they might potentially be good therapeutic targets. To date, the suitability of KIR as a target of intervention has been suggested by studies of bone marrow transplantation [10-12]. Exploitation of NK alloreactivity may become an important therapeutic strategy in the management of myeloid malignancy, in the modulation of engraftment procedures and in the control of graft-versus-host-disease [13,14]. In autoimmune disease, NK cells can promote or inhibit the activation of autoimmune T cells, and by virtue of their ability to rapidly kill abnormal cells and produce cytokines and chemokines, NK cells play a key role in regulating autoimmune responses. Human studies and mice models suggest that the immunomodulatory role of NK cells in autoimmunity is likely to provide new insights into the pathogenesis and treatment of autoimmune disorders [15].

Because NK cells respond to cytokines, and because their killing activity can be enhanced by the presence of IL-2, some investigators have suggested that adaptive transfer of NK cell subsets in an activated state (after stimulation with IL-2, IL-12 and IL-15) may be necessary for optimal efficacy [16,17]. However, injection of proinflammatory cytokines such as IL-2 or IL-15 to activate endogenous NK cells may induce inflammation and autoreactivity.

Therefore, expansion of NK cells *ex vivo *is a strategy that is worth considering for several clinical applications. It remains to be determined whether the increase in NK cells *ex vivo *after exposure to cytokines modifies their peripheral repertoire. In the mouse, cytokines such as IL-2, IL-15, IL-21 and IL-4 can prompt considerable modifications and selective alterations in the repertoire of murine NK cells [18]. In the present study we assessed the effects of IL-2, IL-15 and IL-21 on the human NK cell repertoire, and our findings indicate that addition of IL-21 to IL-2 or IL-15 induced a marked increase in NK cell numbers, and that IL-2 and IL-15 may induce the expression of KIR receptors in a KIR-negative fraction. Our data also indicate that IL-21 can downregulate the expression of NKp44 receptor via the adaptor DAP-12 while preserving production of cytokines by the CD56^bright ^and CD56^dim ^subpopulations of NK cells and even enhancing their cytotoxic function.

## Materials and methods

### Reagents and cytokines

RPMI-1640 medium, and β-mercaptoethanol were purchased from Sigma Chemicals (St. Louis, MO, USA). Phosphate-buffered saline (PBS), penicillin/streptomycin, L-glutamine, minimal essential medium nonessential amino acids, and sodium pyruvate were supplied by Gibco Invitrogen (San Diego, CA, USA). Human AB serum was provided by the Blood Bank of Geneva University Hospital (Geneva, Switzerland). Ficoll-Paque™ Plus was from Amersham Biosciences (Uppsala, Sweden). Human recombinant (rh)IL-2 was obtained from Biogen Inc. (Cambridge, MA, USA), rhIL-15 was kindly provided by Invitrogen (Seattle, WA, USA) and rhIL-21 was a gift from Dr DC Foster (Zymogenetics, Seattle, WA, USA).

### Isolation of NK cells, of CD56^dim ^and CD56^bright ^subpopulations, and cell cultures

Peripheral blood mononuclear cells (PBMCs) were isolated from normal young donors by density-gradient centrifugation. NK cells were separated from 300 × 10^6 ^PBMCs by negative selection using an isolation kit (Miltenyibiotec, Bergisch Gladbach, Germany). Non-NK cells from human PBMCs, such as T cells, B cells, dendritic cells, monocytes, granulocytes and erythrocytes, were stained with a cocktail of biotin-conjugated antibodies to CD3, CD4, CD14, CD15, CD19, CD36, CD123 and CD123a. A second staining was conducted using an antibiotin mAb conjugated with microbeads. The NK cells were isolated by depletion of the magnetically labelled cells. After negative selection, between 3% and 10% NK cells were recovered (depending on the donor). NK cells were washed with PBS and stained with APC-conjugated mAb to CD56 (Miltenyibiotec), biotin-conjugated mAb to CD16 (BD Pharmingen™, San Diego, CA, USA) and APC-Cy7-conjugated mAb to CD3 (BD Pharmingen™). The unlabeled CD16 mAb was stained with streptavidin-ECD, a tandem dye comprising PE covalently linked to Texas-Red (BD Pharmingen™). CD56^dim ^and CD56^bright ^NK cells were subsequently separated on a FACSAria^® ^sorter (BD Pharmingen™). The purity of each subpopulation was consistently greater than 95%. The selected NK cells were cultured at a concentration of 1 × 10^6 ^cells/ml for up to 7 days in RPMI medium supplemented with the following: 10% HI AB serum, 100 U/ml penicillin, 100 μg/ml streptomycin, 2 mmol/l L-glutamine, 1% minimal essential medium nonessential amino acids and 0.1 mmol/l sodium pyruvate, 5 mmol/l β-mercaptoethanol (at 5 × 10^-5 ^mol/l; referred to as 'medium'). Then, rhIL-2 (25 ng/ml), rhIL-15 (25 ng/ml), rIhL-2 plus rhIL-21 (50 ng/ml), or rhIL-15 plus rhIL-21 was added. The NK cells were placed in 5% carbon dioxide-air humidified atmosphere at 37°C.

### Cytokine determination

Samples of conditioned medium were subjected to enzyme-linked immunosorbent assay for determination of IFN-γ. The sensitivity of all protein assays was 10 to 30 pg/ml. In addition to enzyme-linked immunosorbent assay, an IFN-γ capture assay kit (Miltenyibiotec) was used to determine the amount of IFN-γ. After 7 days of culture with different cytokines, CD56^dim ^and CD56^bright ^NK cells were incubated for 45 min at 37°C together with a bipolar anti-IFN-γ antibody, which binds to the cells as well as to IFN-γ secreted on the cell surface. By using a second antibody, namely PE-conjugated anti-IFN-γ, IFN-γ was determined by fluorescence-activated cell sorting (FACS).

### Cell staining for flow cytometry

The following mouse anti-human mAbs were purchased from BD Pharmingen™: PE-Cy7-conjugated anti-CD3; APC-Cy7-conjugated anti-CD16; FITC-conjugated anti-CD158b, which recognizes KIR2DL2 (CD158b1), KIR2DL3 (CD158b2) and KIR2DS2 (CD158j); PE-conjugated anti-CD158a specific for KIR2DL1 (CD158a) and KIR2DS1 (CD158h); biotin-conjugated anti-NKB1 specific for KIR3DL1 (CD158e1); and APC-conjugated anti-NKG2D. PE-conjugated anti-CD56, PE-conjugated anti-CD158i (KIR2DS4) and PE-conjugated anti-NKG2A were supplied by Beckmann Coulter (Fullerton, CA, USA). APC-conjugated anti-CD56, PE-conjugated anti-NKp46, PE-conjugated anti-NKp44 and biotin-conjugated anti-Nkp30 were from Miltenyibiotec, and PE-conjugated anti-NKG2C was from R&D (R&D Systems Inc., Minneapolis, MN, USA). The unlabeled NKB1 mAb was stained with streptavidin-ECD (Beckmann Coulter). For indirect immunofluorescence, nonspecific binding sites were saturated with normal mouse serum before adding the relevant mAb. Six-colour immunofluorescence was performed to assess surface marker expression on CD56^dim ^and CD56^bright ^NK cells activated by rhIL-2, rhIL-15, rhIL-2 plus rhIL-21, or rhIL-15 plus rhIL-21. After 7 days of culture, CD56^dim ^and CD56^bright ^NK cells were washed twice with PBS (completed with 2% foetal calf serum [FCS]) and treated successively with FITC-, PE-, biotin-streptavidin-ECD-, APC-, APC-Cy7-, and PE-Cy7-conjugated mAbs on ice for 10 minutes and washed with PBS (completed with 2% FCS). For the capture assay, NK cells were isolated and washed once with PBS, complemented with 2% FCS. IFN-γ was determined using the capture assay kit from Miltenyibiotec, in accordance with supplier's instructions. Cell staining was analysed using FACSAria^® ^and FACS DIVA™ software (BD Pharmingen™).

### CFSE labelling and analysis of NK cell proliferation *in vitro*

A total of 400 × 10^6 ^human PBMCs were labelled with fluorochrome 5-carboxyfluorescein diacetate succinimidyl ester (CFSE; Molecular Probe, Inc., Portland, OR, USA), as described previously [19]. CFSE was dissolved in dimethyl sulfoxide and added to the cell suspension for 15 minutes at a final concentration of 0.5 μmol/l at 37°C. The reaction was stopped by the addition of PBS/10% FCS. The cells were washed in PBS/10% FCS, resuspended in RPMI and left to rest overnight at 37°C in a 5% CO_2 _humid atmosphere. The next day NK cells were isolated from 300 × 10^6 ^PBMCs (stained with CFSE) using the NK cell isolation kit (see 'Isolation of NK cells, of CD56^dim ^and CD56^bright ^subpopulations, and cell cultures', above). Then, CD56^dim ^and CD56^bright ^NK cells were separated on a FACSAria^® ^sorter (BD Biosciences PharMingen). The isolated CD56^dim ^and CD56^bright ^NK cells were resuspended in RPMI and cultured at 1 × 10^6 ^cells/ml with rhIL-2 (25 ng/ml), rhIL-15 (25 ng/ml), rhIL-2 plus rhIL-21 (50 ng/ml), or rhIL-15 plus rhIL-21 for 5 and 7 days. On days 5 and 7, cells were stained with several conjugated antibodies (see 'Cell staining for flow cytometry', above) and six-colour analysis by flow cytometry was performed on a Becton-Dickinson FACSAria^® ^equipped with FACS DIVA™ software. Live events were collected and analysed by gating on to CD56^dim ^or CD56^bright ^CFSE-positive cells.

### Calculation of the frequency of proliferating NK cells

Proliferation of NK cells in response to cytokine stimulation was analyzed as described previously [19]. By means of the FACS acquisition software (FACS DIVA™), the total number of cells in each generation of proliferation was calculated and the number of precursors generating the daughter cells was determined using the formula *y*/2*n*, where *y *is the number of cells in each peak and *n *is the number of cell divisions. The frequency of NK cell proliferation was then calculated by dividing the total number of precursors by the total number of CFSE-labelled cells.

### Cytotoxicity assay

The isolated NK cells were cultured with rhIL-2 (25 ng/ml), rhIL-15 (25 ng/ml), rhIL-2 plus rhIL-21 (50 ng/ml), or rhIL-15 plus rhIL-21 for 7 days. On day 7, NK cells were subjected to the cytotoxicity assay. To test cytotoxicity, a standard ^51^Cr-release assay was performed. K562 cells were incubated for 1 hour with Na_2 _^51^CrO_4 _(Hartmann Analytics, Braunschweig, Germany), washed three times and co-incubated for 4 hours with NK effector cells. The percentage of specific lysis was calculated from the following formula: percentage of specific lysis = ([experimental counts – spontaneous lysis]/[maximal lysis – spontaneous lysis] × 100). Experiments were conducted in triplicate.

### RNA isolation and real-time PCR

Total cellular RNA was isolated from NK cells by lysing the cells with Qiagen reagent and Qiagen Rneasy^® ^Micro Kit (Qiagen AG, Basel, Switzerland), in accordance with the manufacturer's instructions. One microgram of RNA was treated with DNase to eliminate any contaminating genomic DNA and subsequently reverse transcribed. The quality of the reverse transcription was tested for the expression of the housekeeping gene 18S using real-time polymerase chain reaction (PCR). Subsequently, the relative abundance of KIR genes was determined by TaqMan real-time PCR analysis on an ABI Prism 7300 Sequence detection instrument (Applied Biosystems, Forster City, CA, USA). To quantify the levels of cDNA, the expression of DAP12 and DAP10 was normalized against the housekeeping gene 18S. Data were expressed as relative fold difference between cDNA of the study samples (DAP12 at day 7 with IL-15 and IL-15/IL-21) and a calibrated sample (DAP12 at day 0). DAP12 (Hs00182426_m1), DAP10 (Hs00367159_m1) and 18S (4310893E) primer-probe sets were purchased from Applied Biosystems (Foster City, CA, USA).

### Western blot analysis

Purified NK cells were cultured for 7 days with IL-15 (25 ng/ml) or IL-15 plus IL-21 (50 ng/ml). On day 7, cells were harvested and resuspended at 4 × 10^6 ^cells/ml in 800 ml of ice-cold PBS and centrifuged. Total cell lysate was prepared and subjected to Western blot analysis. The blots were probed with anti-DAP12 (Santa Cruz Biotechnology, Santa Cruz, CA, USA) and anti-β-tubulin (Sigma). A secondary horseradish peroxidase-conjugated goat anti-rabbit (Dako, Glostrup, Denmark) was added for detection. Antibody-bound proteins were detected by the Uptilight hrp Blot Chemiluminescence substrate (Uptima, Montluçon, France).

### Statistical analysis

Data were analyzed using two-factor analysis of variance test. *P *< 0.05 and were considered significant (Statview 5.1 [SAS Institute Inc., Cary, NC, USA] and GraphPad Prism 3.02 [GraphPad, Witzenhausen, Germany]).

## Results

### IL-21 acts in synergy with IL-2 or IL-15 to increase NK cell subpopulations

**Figure 1:**
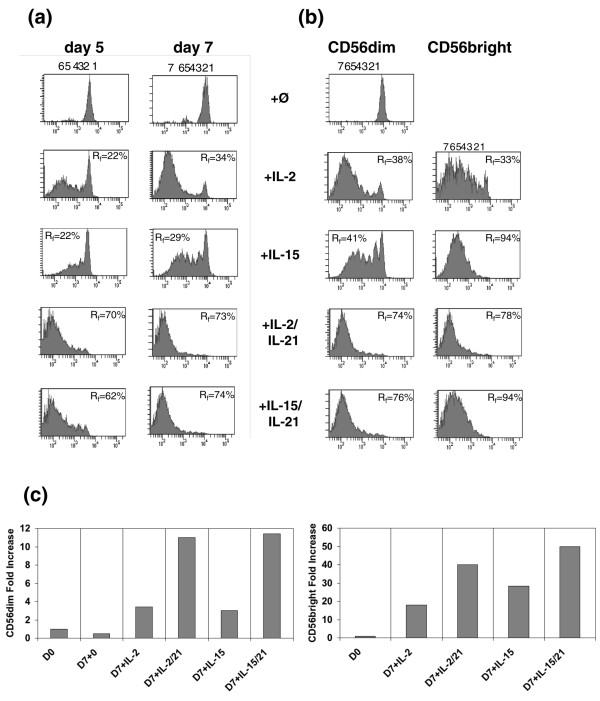
*Ex vivo *NK proliferation with IL-2 or IL-15 in the presence or absence of IL-21. **(a) **Proliferation of bulk natural killer (NK) cells. NK cells stained with CFSE (5-carboxyfluorescein diacetate succinimidyl ester) were gated onto the CD3-negative fraction from peripheral blood mononuclear cells and cultured for seven days with IL-2 or IL-15 in the presence or absence of IL-21. The number of cells undergoing division was analyzed at days 5 and 7. Proliferating NK cells are expressed in percentages. This experiment is representative of three individual experiments performed. **(b) **Proliferation of NK cell subpopulations. NK cells stained with CFSE were purified by magnetic beads, and CD56^dim ^and CD56^bright ^subpopulations were isolated by fluorescence-activated cell sorting. The two populations were cultured for 7 days with IL-2 or IL-15 in the presence or absence of IL-21 and analyzed at day 7. The percentage indicates proliferating NK cells. This experiment is representative of three individual experiments performed. **(c) **Amplification of CD56^dim ^and CD56^bright ^subpopulations. Sorted 200,000 CD56^dim ^and CD56^bright ^NK cells were plated at day 0 and cultured for 7 days with IL-2 or IL-15 in the presence or absence of IL-21. At day 7 NK cells were stained for their purity (data not shown) and counted. The results are expressed as fold increase as compared to day 0.

To investigate the function of IL-21 in subpopulations of human NK cells, we purified mature human NK cells by negative selection (Miltenyi beads; Figure 1a). CFSE-labelled primary NK cells and both CD56^bright ^and CD56^dim ^populations were then cultured for up to 7 days in the presence of IL-2 or IL-15 with or without the addition of IL-21 (Figure 1a,b). At days 5 (Figure 1a [left panel]) and 7 (Figure 1a [right panel]), addition of IL-21 to each IL-2 and IL-15 culture resulted in a marked increase in cell division as compared with IL-2 and IL-15 alone. As previously reported [20], IL-21 alone does not induce NK cell proliferation (data not shown). In the next step, CD56^bright ^and CD56^dim ^subpopulations were purified by FACS to elucidate the role played by IL-21 in the expansion of the NK cell subpopulations in the presence of IL-2 and IL-15 (Figure 1b). At day 7, IL-21 acted synergistically with IL-2 or IL-15 in expanding the CD56^dim ^population (Figure 1b [left panel]). IL-15 alone proved sufficient to increase markedly the CD56^bright ^population, whereas IL-21 induced a significant increase in the CD56^bright ^population with IL-2 (Figure 1b [right panel]). The increase in division mediated by IL-2 and IL-15 plus IL-21 was matched by an increase in total cell number of NK cells. We observed a 10-fold increase in cell numbers with IL-2/IL-21 or IL-15/IL-21 on the CD56^dim ^population, as compared with 3-fold and 2.8-fold increases with IL-2 and IL-15, respectively. The expansion was more efficient on the CD56^bright ^population (an 18-fold and 28-fold increase with IL-2 or IL-15 alone, as compared with a 40-fold and 50-fold increase with IL-2/IL-21 and IL-15/IL-21, respectively; Figure 1c).

### The KIR repertoire of NK subpopulations after seven days of culture with IL-2 and IL-15 in the presence or absence of IL-21

**Figure 2:**
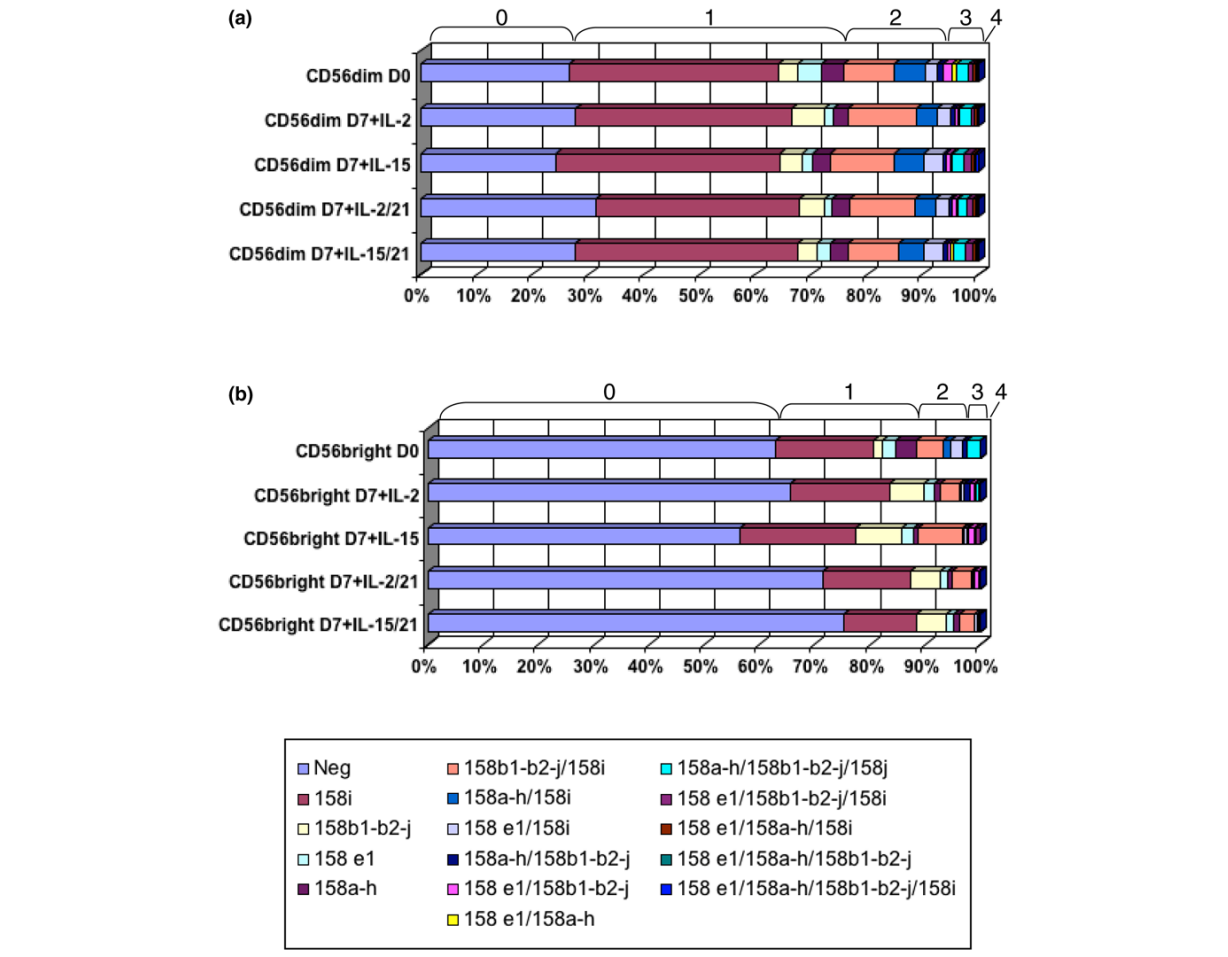
Expression of KIR repertoire after culture *ex vivo *of NK cells with IL-2 or IL-15 with/without IL-21. **(a) **Expression of different killer cell immunoglobulin-like receptor (KIRs) on the CD56^dim ^natural killer (NK) population. NK cells were purified by magnetic beads, and the CD56^dim ^subpopulations were isolated by fluorescence-activated cell sorting (FACS) and cultured for seven days with IL-2 or IL-15 in the presence or absence of IL-21. Each bar represents different culture conditions, and within the bar are shown the percentages of expression of KIR combinations: no KIR, and one, two, three, or four KIR combinations. KIR combination signifies KIR recognized by a single antibody. Anti-CD158a recognizes KIR2DL1 (CD158a) and KIR2DS1 (CD158h). Anti-CD158b recognizes KIR2DL2 (CD158b_1_), KIR2DL3 (CD158b_2_) and KIR2DS2 (CD158j). Anti-NKB1 is specific for KIR3DL1 (CD158e_1_), and anti-KARp50.3 (CD158i) recognizes KIR2DS4. This experiment is representative of five individual experiments performed. **(b) **Expression of the different KIR receptors on the CD56^bright ^NK population. NK cells were purified by magnetic beads and the CD56^bright ^subpopulations were isolated by FACS sorting and cultured for 7 days with IL-2 or IL-15 in the presence or absence of IL-21. Each bar represents different culture conditions, and within the bar are shown the percentages of expression KIR combinations: no KIR, and one, two, three, or four KIR combinations. KIR combination signifies KIR recognized by a single antibody. Anti-CD158a recognizes KIR2DL1 (CD158a) and KIR2DS1 (CD158h). Anti-CD158b recognizes KIR2DL2 (CD158b_1_), KIR2DL3 (CD158b_2_) and KIR2DS2 (CD158j). Anti-NKB1 is specific for KIR3DL1 (CD158e_1_), and anti-KARp50.3 (CD158i) recognizes KIR2DS4. This experiment is representative of five individual experiments performed.

To analyze further the expression of the KIR repertoire on CD56^bright ^and CD56^dim ^NK cell populations, we assessed the expression of each KIR receptor on samples from five normal donors before and after 7 days of culture with IL-2 and IL-15 alone or IL-2/IL-21 and IL-15/IL-21. Figure 2 and Table 1 depict a typical example of KIR 2DL1/S1 (anti-CD158a), KIR 2DL2/L3/S2 (anti-CD158b), KIR 3DL1 (anti-CD158e1) and KIR 2DS4 (anti-CD158i) from a normal donor, detected at the cell surface by multi-colour cytofluorometry. Because the antibodies available failed to distinguish every activating and inhibitory KIR (with the exception of 2DS4 and 3DL1), or even 2DL2 and 2DL3, the KIR and KIR combinations recognized by each antibody are discussed below. Of the CD56^dim ^NK cell population, 26% did not express any KIR at their cell surface at day 0. 2DS4 was expressed by 37.5% of CD56^dim ^NK cells and only around 4% of the cells expressed at least one of the other KIR combinations (2DL1/S1, 2DL2/L3/S2, or 3DL1). Of CD56^dim ^cells 20.21% expressed at least two KIR combinations, mostly 2DS4 associated with 2DL2/L3/S2 (9.04%), 2DS4 with 2DL1/S1 (5.6%), and 2DS4 with 3DL1/S1 (2.24%) in this typical example. A very small fraction of CD56^dim ^expressed two KIR combinations without 2DS4 (0.80% to 1.64%). Three KIR combinations were expressed in 3.83% of CD56^dim^, mostly 2DS4 plus 2DL1/S1 plus 2DL2/L3/S2 (2.32%), and only 0.17% expressed four KIR receptor combinations at the cell surface. After 7 days of culture with IL-2 or IL-15 and IL-2/IL-21 or IL-15/IL-21, the KIR repertoire had not undergone any significant modification and had remained virtually stable regardless of cytokine regimen, but the percentage of KIR-negative cells tended to increase in the presence of IL-21 (Figure 2a and Table 1).

**Table 1:** The KIR phenotype on NK cells stimulated with IL-2, Il-15 and IL-21.

	Day 0	Day 7 + IL-2	Day 7 + IL-15	Day 7 + IL-2/IL-21	Day 7 + IL-15/IL-21
CD56^dim^					
Negative	26.63	27.77	24.16	31.28	27.66
158i	37.57	38.81	40.33	36.53	40.00
158b1-b2-j	3.46	5.81	3.97	4.46	3.41
158e1	4.16	1.45	1.83	1.46	2.30
158a-h	3.97	2.71	3.22	3.23	3.27
158b1-b2-j/158i	9.04	12.29	11.28	11.57	8.92
158a-h/158i	5.60	3.77	5.37	3.76	4.71
158e1/158i	2.24	2.24	3.41	2.39	3.29
158a-h/158b1-b2-j	0.89	0.97	0.72	0.67	0.78
158e1/158b1-b2-j	1.64	0.35	0.72	0.61	0.66
158e1/158a-h	0.80	0.32	0.36	0.30	0.50
158a-h/158b1-b2-j/158i	2.32	2.06	2.02	1.75	2.06
158e1/158b1-b2-j/158i	0.68	0.63	1.31	0.96	1.30
158e1/158a-h/158i	0.42	0.50	0.66	0.50	0.70
158e1/158a-h/158b1-b2-j	0.41	0.10	0.22	0.21	0.14
158e1/158a-h/158b1-b2-j/158i	0.17	0.18	0.41	0.32	0.30
CD56^bright^					
Negative	62.86	65.69	56.57	71.55	75.24
158i	17.86	17.89	20.87	15.75	13.20
158b1-b2-j	1.67	6.11	8.34	5.45	5.48
158e1	2.38	1.87	2.17	1.29	1.24
158a-h	3.81	1.20	0.77	0.86	1.16
158b1-b2-j/158i	4.76	3.44	8.01	3.56	2.54
158a-h/158i	1.19	0.33	0.28	0.19	0.20
158e1/158i	2.38	0.62	0.51	0.22	0.36
158a-h/158b1-b2-j	0.71	1.00	0.23	0.15	0.12
158e1/158b1-b2-j	0.00	0.89	1.22	0.65	0.00
158e1/158a-h	0.00	0.13	0.10	0.00	0.16
158a-h/158b1-b2-j/158i	2.38	0.56	0.21	0.11	0.06
158e1/158b1-b2-j/158i	0.00	0.22	0.70	0.22	0.20
158e1/158a-h/158i	0.00	0.04	0.02	0.00	0.04
158e1/158a-h/158b1-b2-j	0.00	0.00	0.00	0.00	0.00
158e1/158a-h/158b1-b2-j/158i	0.00	0.00	0.00	0.00	0.00

Anti-CD158a recognize KIR2DL1 (CD158a) and KIR2DS1 (CD158h). Anti-CD158b recognize KIR2DL2 (CD158b1), KIR2DL3 (CD158b2) and KIR2DS2 (CD158j). Anti-NKB1 is specific for KIR3DL1 (CD158e1). Anti-KARp50.3 recognize KIR2DS4 (CD158i). KIR, killer cell immunoglobulin-like receptor.

As expected, a higher percentage of CD56^bright ^NK cells failed to express KIR at the cell surface (62.9%) [6]. Of CD56^bright ^NK cells, 17.9% expressed 2DS4 only and around 7.86% of CD56^bright ^expressed at least one of the other KIR (2DL1/S1, 2DL2/L3/S2, or 3DL1) only. 2DS4 and one additional KIR were expressed in 8.33% of the CD56^bright ^cells. 2DS4 plus 2DL1/S1 plus 2DL2/L3/S2 were expressed in 2.38% of the cells, and none of the other combinations of three or four KIR was found at day 0. After 7 days of culture with the different cytokines, the percentage of cells lacking KIR expression increased with IL-21: 62.9% at day 0, 65% and 56% at day 7 with IL-2 and IL-15, respectively, versus 71.55% and 75.24% at day 7 with IL-2/IL-21 and IL-15/IL-21, respectively. Expression by CD56^bright ^cells of two or more KIRs fluctuated more than that of CD56^dim ^(Figure 2b and Table 1), but considering the small percentages of these populations we have refrained from drawing definitive conclusions from these results.

Several donors were tested, and each time between 25% to 45% of CD56^dim ^cells failed to express any KIR, and 35% to 45% expressed predominantly one type of KIR (for example, 2DS4 in Figure 2). In CD56^bright ^between 60% and 85% of the cells did not express any KIR. We did not observe a selective induction of KIR receptors at the cell surface of CD56^bright ^and CD56^dim ^NK cells after 7 days of culture with IL-2 and IL-15 alone or IL-2/IL-21 and IL-15/IL-21, but the fraction of NK cells without any KIR at their surface tended to increase in the presence of IL-21, especially on CD56^bright ^NK cells.

### IL-2 and IL-15 induce KIR expression on KIR negative population

It is generally thought that the KIR receptors are acquired by a stochastic mechanism, currently poorly understood, which operates exclusively during NK cell development, and that the repertoire is fixed after maturation. However, in mouse, Gays and coworkers [18] showed that expression of the Ly49 receptor (the mouse equivalent of KIR in the human system [18]) can be regulated by cytokines on mature NK cells.

**Figure 3:**
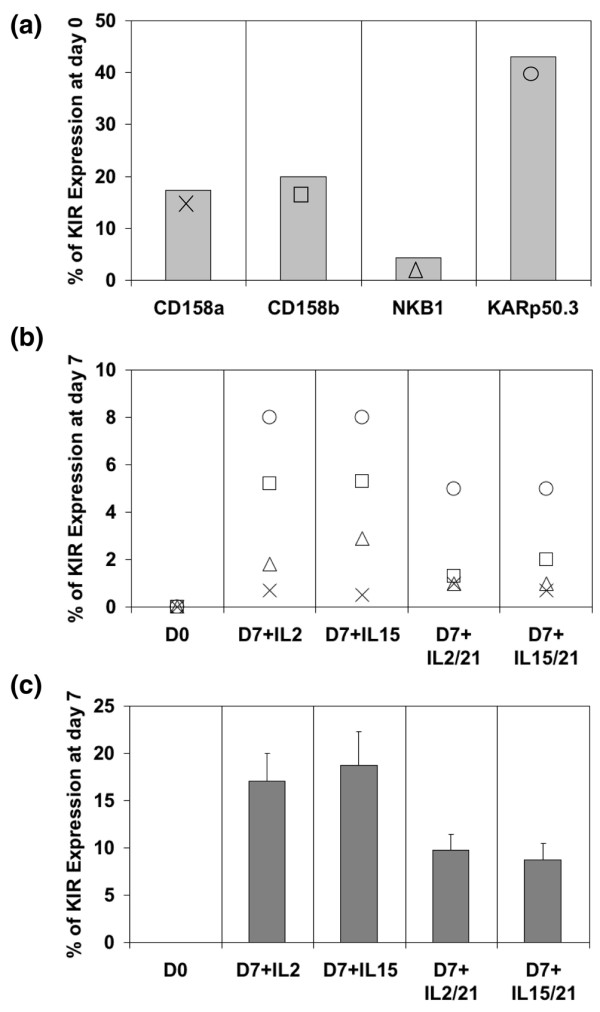
KIR expression in a population of KIR-negative NK cells. **(a) **Killer cell immunoglobulin-like receptor (KIR) repertoire of a prototypical blood donor at day 0. The KIR repertoire was assessed on the natural killer (NK) bulk population at day 0 before the sorting of the KIR-negative fraction. Anti-CD158a recognizes KIR2DL1 (CD158a) and KIR2DS1 (CD158h). Anti-CD158b recognizes KIR2DL2 (CD158b_1_), KIR2DL3 (CD158b_2_) and KIR2DS2 (CD158j). Anti-NKB1 is specific for KIR3DL1 (CD158e_1_), and anti-KARp50.3 (CD158i) recognizes KIR2DS4. This experiment is representative of three individual experiments performed. **(b) **KIR repertoire of the KIR-negative population after 7 days of culture. The KIR-negative population of the same donor (panel a) was selected by fluorescence-activated cell sorting (FACS; left column), and cultured for 7 days with IL-2 or IL-15 in the presence or absence of IL-21 (the four columns to the right). The KIR repertoire was assessed after 7 days of culture. Anti-CD158a recognizes KIR2DL1 (CD158a) and KIR2DS1 (CD158h). Anti-CD158b recognizes KIR2DL2 (CD158b_1_), KIR2DL3 (CD158b_2_) and KIR2DS2 (CD158j). Anti-NKB1 is specific for KIR3DL1 (CD158e_1_), and anti-KARp50.3 (CD158i) recognizes KIR2DS4. This experiment is representative of three individual experiments performed. The symbols used are defined in panel a. **(c) **Effect of IL-2 and IL-15 and/or IL-21 on the KIR repertoire of several donors. The KIR-negative population was selected by FACS sorting (left column) and cultured for 7 days with IL-2 or IL-15 in the presence or absence of IL-21. This experiment represents the fraction of KIR-negative sorted cells from five normal donors who expressed KIRs after 7 days.

To determine whether KIR would be expressed at the cell surface of mature human NK cells, we focused more directly on the KIR-negative population. We eliminated the KIR-positive fraction by cell sorting and cultured the KIR negative fraction in the presence of IL-2, IL-15, with or without IL-21. Figure 3a,b is representative of three separate experiments. Figure 3a shows the KIR repertoire of a normal donor. In Figure 3b, we show the purity of the KIR-negative population after the depletion of the KIR-positive population by FACS sorting for the same donor (left column). The following right column show the expression of the KIR repertoire in a fraction of the KIR-negative cells after 7 days of culture with IL-2 and IL-15 with or without IL-21. For every donor tested, 15% to 20% of the KIR-negative fraction expressed KIR receptors at their cell surface after 7 days of culture with IL-2 and IL-15 (Figure 3c). Interestingly, the addition of IL-21 partially inhibited the expression of KIR receptors (Figure 3b,c).

**Figure 4:**
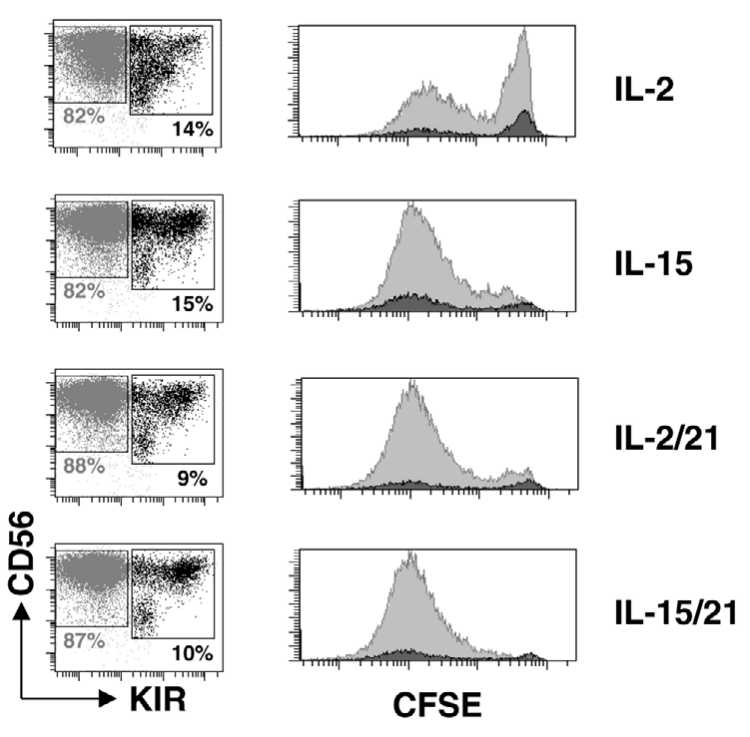
Proliferation of KIR-negative and KIR-positive NK cells. Peripheral blood mononuclear cells were stained with CFSE (5-carboxyfluorescein diacetate succinimidyl ester) and natural killer (NK) cells were purified by magnetic beads. The CFSE-positive, killer cell immunoglobulin-like receptor (KIR)-negative fraction was purified by fluorescence-activated cell sorting before culture for 7 days with IL-2 or IL-15 in the presence or absence of IL-21. The grey dots represent the KIR-negative fraction and the black dots represent the KIR-positive fraction after 7 days of culture (left panels). The percentage indicates the fraction KIR-positive and KIR-negative NK cells after 7 days of culture. The number of cells undergoing division in the KIR negative (in grey) and the KIR-positive (in black) fraction were analyzed after 7 days of culture on CD56^+^/KIR^- ^(in grey) or CD56^+^/KIR^+ ^(black) gated cells (right panel).

To rule out the possibility of a proliferation of a small residual fraction of KIR-positive receptors, the NK cells were stained with CFSE and the KIR-negative fraction was sorted by FACSAria^®^. After 7 days of culture we noted that the NK cell population expressing the KIR receptor (Figure 4 [black histogram]) underwent division to smaller extent than the KIR-negative population (Figure 4 [grey histogram]), demonstrating that the KIR-positive NK cell population does not possess a proliferative advantage. These results strongly suggest that KIR receptors may be expressed *de novo *in the presence of IL-2 or IL-15 in mature human NK cells.

### NKp44 expression is induced by IL-2 and IL-15 and down-regulated by IL-21

**Figure 5:**
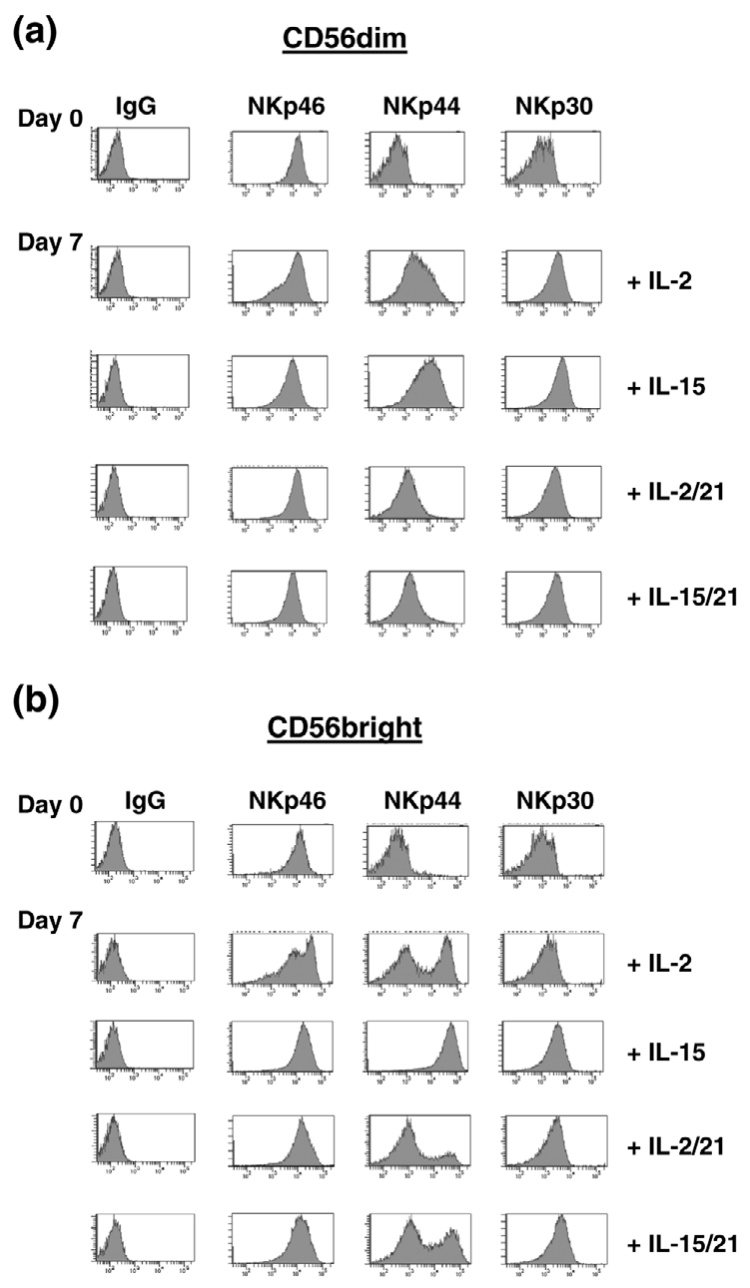
NCR repertoire expressed after culture *ex vivo *of NK cells with IL-2 or IL-15 with-without IL-21. **(a) **Expression of the natural cytotoxicity receptor (NCR) family on the CD56^dim ^population. Natural killer (NK) cells were purified by magnetic beads, and the CD56^dim ^subpopulation was isolated by fluorescence-activated cell sorting (FACS) and cultured for 7 days with IL-2 or IL-15 in the presence or absence of IL-21. NKp46, NKp44 and NKp30 were assessed on the CD56^dim ^population at days 0 and 7. One of three similar experiments is shown. **(b) **Expression of the NCR receptor family, NKp46, NKp44 and NKp30 on the CD56^bright ^population. NK cells were purified by magnetic beads and the CD56^bright ^subpopulations were isolated by FACS sorting and cultured for 7 days with IL-2 or IL-15 in the presence or absence of IL-21. NKp46, NKp44 and NKp30 were assessed on the CD56^bright ^population at days 0 and 7. One of three similar experiments is shown.

NCR receptors (NKp46, NKp44 and NKp30) are known to mediate cytotoxicity to a variety of tumour target cells but also to pathogen-specific antigens. Even though their ligands are unknown, they appear to play a crucial role by activating NK cells in the absence of additional stimuli [21]. NKp46 was expressed by 100% of the CD56^bright ^and CD56^dim ^NK cell populations and was not modified after 7 days of culture in the presence of cytokines (Figure 5a,b [left]). NKp30 was also expressed in 100% of the cells but to a lesser extent and it was upregulated in both CD56^bright ^and CD56^dim ^NK cell populations in the presence of IL-2, IL-15 and IL-21 (Figure 5a,b [right]). Although NKp44 was not expressed by fresh primary NK cells, it was expressed by both populations upon activation by cytokines as previously described [22]. According to our data, IL-2 and IL-15 induced NKp44 expression in 100% of both NK populations (Figure 5a,b [centre]). The addition of IL-21 to IL-2 or IL-15 downregulated NKp44 expression on CD56^dim ^and CD56^bright ^NK cells (Figure 5a,b [centre]). Interestingly, a fraction of CD56^bright ^NK cells appeared to be resistant to downregulation by IL-21 (Figure 5b [centre]) because 20% and 40% of cells treated with IL-2/IL-21 and IL-15/IL-21, respectively, continued to express NKp44. The NKp44 receptor mediates signal transduction through the association of adaptor molecules, in particular DAP12 in humans [23]. Given the importance of DAP12 in NKp44 expression, we conducted real-time PCR and Western blot to analyze the expression of DAP12.

**Figure 6:**
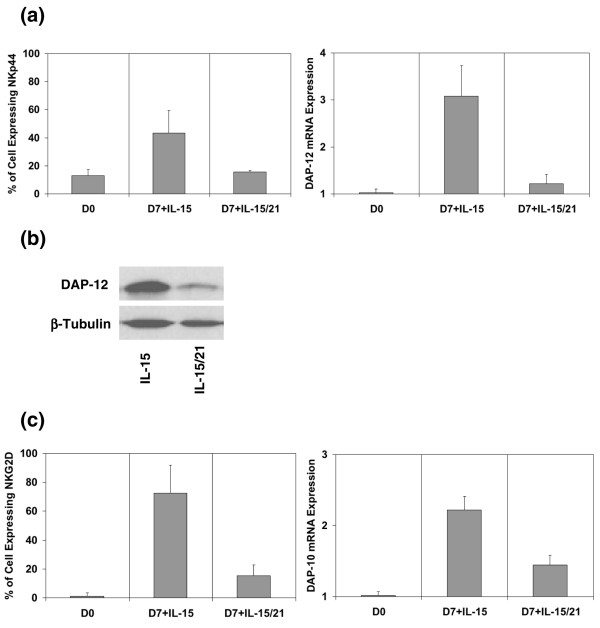
IL-21 downregulated cell surface expression of NKp44 and NKG2D and expression of DAP10 and DAP12. **(a) **IL-21 downregulated NKp44 and DNAX-activating protein of 12 kDa (DAP12). The percentage of natural killer (NK) cells expressing NKp44 at the cell surface after addition of IL-21 to IL-15 (left) was diminished. Simultaneously, transcripts of the adaptor DAP12 were quantified by real-time PCR after 7 days of culture with IL-15 and compared with the same culture conditions when IL-21 was added (right). The results indicate a reduction in DAP12 transcripts after addition of IL-21 compared with day 0. The expression of DAP12 cDNA was normalized against the housekeeping gene 18S. Mean ± standard deviation of three experiments is shown. **(b) **IL-21 downregulated DAP12 at the protein level. NK cells were purified by magnetic beads and cultured with IL-15 in presence or absence of IL-21. After 7 days of culture, the adaptor DAP12 protein was quantified by Western blot. The blots were probed with anti-DAP12 and anti-β-tubulin antibodies. **(c) **IL-21 downregulated NKG2D and DAP10. The percentage of NK cells expressing NKG2D at the cell surface after addition of IL-21 to IL-15 (left) is shown. Simultaneously, the adaptor DAP10 transcripts were quantified by real-time PCR after 7 days of culture with IL-15 and compared with the same culture condition when IL-21 was added (right). As previously shown by Burgess and coworkers [24], in the presence of IL-2, IL-21 down-regulated the expression of C-lectin receptor NKG2D at the cell surface and the transcript of the adaptor DAP10 after 7 days of culture with IL-15. The expression of DAP10 cDNA was normalized against the housekeeping gene 18S. Mean ± standard deviation of three experiments is shown.

As shown in Figure 6a (left), the cell surface expression of NKp44 was markedly reduced after 7 days of culture with IL-15 and IL-21. In the same conditions DAP12 mRNA was strongly reduced (Figure 6a [right]) and a 60% reduction in DAP12 protein level after correction with the β-tubulin was observed (Figure 6b). In the same experiment we confirmed by FACS and real-time PCR that IL-21 downregulates NKG2D and DAP10, respectively [24] (Figure 6c [left and right]). These data suggest that IL-21 regulates both adaptors DAP10 and DAP12, leading to reductions in expression of NKG2D and NKp44.

### NK cell subpopulations produce interferon-γ and mediate cytotoxicity after proliferation *ex vivo *in the presence of cytokines

**Figure 7:**
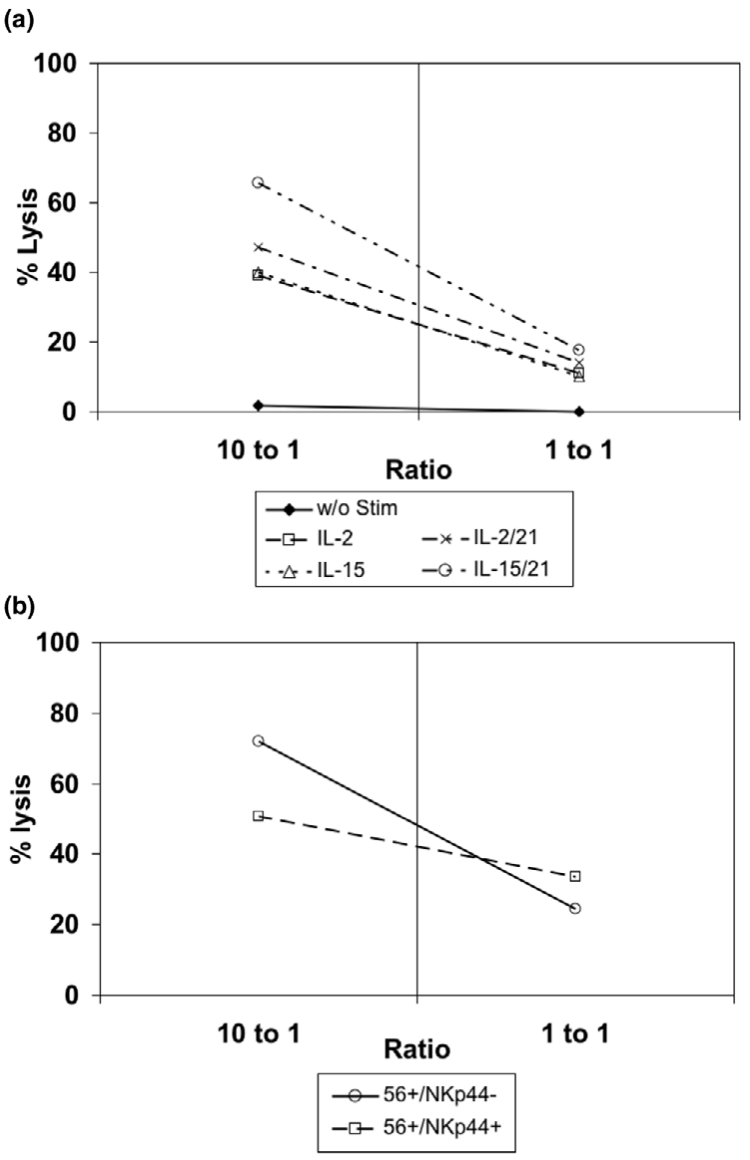
Cytotoxicity of NK cells after *ex vivo *proliferation in the presence of cytokines. **(a) **Cytotoxicity of natural killer (NK) cells to K562 cells. Human primary NK cells were purified by magnetic beads and cultured for 7 days with IL-2 or IL-15 in the presence or absence of IL-21. Both populations were analyzed for cytoxicity to K562 target cells at ratios of 10:1 and 1:1. One of three similar experiments is shown. **(b) **Cytotoxicity of NKp44-positive and NKp44-negative NK cells. Human primary NK cells were purified by magnetic beads (bulk) and cultured for 7 days with IL-15 and IL-21. The NKp44-positive and NKp44-negative subpopulations were sorted by fluorescence-activated cell sorting and were analyzed for their cytotoxicity to K562 targets cells at ratios of 10:1 and 1:1. One of three similar experiments is shown.

Activation of NK cells *in vivo *is mediated by a variety of signals, leading to cytokine secretion and cytotoxic activity. After the expansion *ex vivo *of NK cells in culture, it is crucial to determine whether NK cells continue to kill target cells in response to stimuli. Thus, NK cells were tested with respect to their cytotoxicity to K562 target cells. Seven days of expansion with the different cytokine regimens did not reduce the cytotoxicity to K562 target cells of NK cell population. The mean cytotoxic activity of three experiments was higher with IL-15 and IL-21, as compared with IL-2 and IL-15 alone at a ratio of 10:1 (*P *= 0.02 and *P *= 0.04, respectively) and at the ratio of 1:1 (*P *= 0.015 and *P *= 0.05, respectively; Figure 7a). Because IL-21 also downregulated NKp44/DAP12, we wished to analyze the cytotoxicity of the NKp44-positive or -negative faction. After day 7 of culture of purified NK cells with IL-15 and IL-21, we separated the NKp44-positive fraction from the NKp44-negative fraction and assessed their cytotoxicity potential. Our data demonstrate that the NKp44/DAP12-negative fraction is significantly more cytotoxic than the positive one at a ratio of 10:1 which was not fully expected [25].

**Figure 8:**
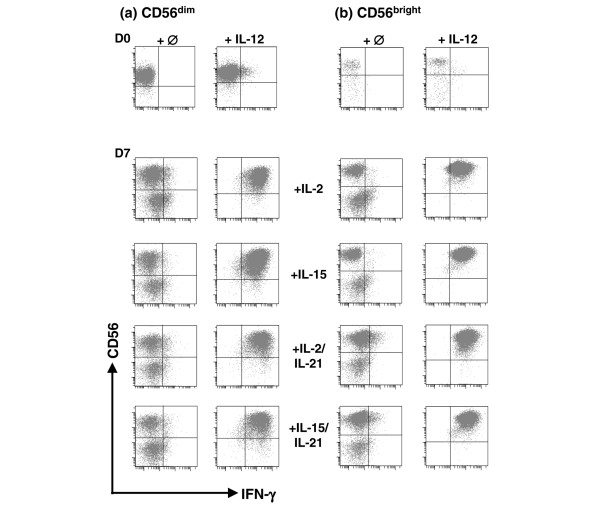
Expression of IFN-γ on NK cells after their proliferation *ex vivo *in presence of cytokines. IFN-γ was analyzed by capture assay on natural killer (NK) cells at day 0 without cytokines, and after 7 days of culture with IL-2 or IL-15 in the presence or absence of IL-21. To determine the capacity of NK cells to produce IFN-γ, cells were subjected to stimulation by IL-12 for 12 hours under all conditions. One of three similar experiments is shown.

Cytokines produced by activated antigen-presenting cells such as IL-12 are potent stimulators of IFN-γ production by NK cells. We therefore tested the IFN-γ production by CD56^dim ^and CD56^bright ^NK cells by IL-12 stimulation after 7 days of culture with IL-2 and IL-15, in addition to IL-21 (Figure 8a,b). Although in the past the production of IFN-γ was considered to be mostly confined to the CD56^bright ^NK cell population [6], our findings show that both populations responded substantially and to similar extents to IL-12 stimulation in terms of IFN-γ production. Moreover, without IL-12, a fraction of NK cells, mostly in the CD56^dim ^population, secreted IFN-γ after 7 days of culture mainly in the presence of IL-21 (Figure 8a,b). The production of IFN-γ detected by FACS capture assay was substantiated by enzyme-linked immunosorbent assay (data not shown).

## Discussion

In the present study, we optimized culture conditions to enhance proliferation of mature human NK cells with IL-2 and IL-15, in the presence or absence of IL-21, and we analyzed the effect that addition of these cytokines had on the NK receptor repertoire of the CD56^dim ^and CD56^bright ^subpopulations of mature NK cells. The main results of this study are as follows. First, IL-21 acts synergistically with IL-2 or IL-15, enhancing markedly the CD56^dim ^NK subpopulation. Second, the KIR repertoire of NK cells was stable in culture, but the KIR-negative cell fraction can express KIR receptors in culture with IL-2 and IL-15, this production being less marked in the presence of IL-21. Finally, IL-21, which is known to downregulate NKG2D, proved also to have the ability to downregulate NKp44 (NCR receptor) induced by IL-15 and to enhance the cytotoxicity of NK cells.

Several experimental studies have demonstrated the capacity of NK cells to eliminate cancer cells. Evidence is now emerging that NK cells might also be a therapeutic target in autoimmunity [2,26]. Two different strategies could be taken into consideration: the activation of endogenous NK cells or their expansion *ex vivo*. Taking into account the effects of IL-2, IL-15 and IL-21 on the differentiation, maturation, proliferation and activation of NK cells [9,27,28], these cytokines would appear to be particularly suited for manipulating NK cells for therapeutic purpose. Several clinical trials have helped to assess the effect of IL-2 administration on activation and expansion of endogenous NK cells [29,30]. Recent reports have revealed the effect of IL-21 in preclinical models, suggesting a strong antitumoural activity of IL-21 in renal cell carcinoma, melanoma and leukaemia [31]. However, all three of IL-2, IL-15 and IL-21 have been implicated in autoimmunity, and using such cytokines to activate endogenous NK cells may favour inflammation and promote autoreactivity [9,27,28].

*Ex vivo *adaptive immunotherapy with NK cells has been tested by several groups that have collected and purified clinical grade NK cells before administering patients with doses of up to 10^7^/kg, and IL-2 has already been used to increase numbers of NK cells in a therapeutic approach to melanoma, renal carcinoma cells, or after haematopoietic stem cell transplantation [32-34]. However, it is supposed that small fractions of NK cells characterized by specific phenotypes are responsible for their antiviral, antitumoural, or immunomodulatory activity. In addition, activation by different stimulus or manipulation of NK cell subpopulations by genetic engineering could be much efficient to design NK-cell based immunotherapeutic strategies [35]. Therefore, starting off with a limited number of NK cells would be of great interest for optimizing protocols for increasing the number of NK cells *ex vivo *by preserving their phenotypes.

Because of their intrinsic effects on NK cells, addition of IL-21 to IL-2 or IL-15 would be the best combination to optimize the *ex vivo *proliferation of NK cells. Recent data demonstrated that cultured NK cells survived better with IL-15 than with IL-2 in the presence of methylprednisolone, offering interesting clues as to an appropriate NK cell cytokine conditioning regimen in adoptive immunotherapy [36]. *In vitro*, the effect of IL-21 on the proliferation and differentiation of murine NK cells proved insufficient to drive the proliferation of immature or naïve NK cells; however, at low doses IL-21 enhanced a proliferative response of these cells to either IL-2 or IL-15, whereas high doses had an inhibitory effect [20]. Interestingly, the number of functional NK cells in the peripheral lymphoid organs of IL-21 receptor null mice and the number of bone marrow NK cell precursors are similar in wild-type and γ c-deficient mice, indicating that γ c-dependent cytokines are not required for the earliest commitment events in the NK cell lineage [37]. In addition, Gays and colleagues [18] demonstrated that IL-21 and combinations of IL-21 and IL-15 or IL-4 can downregulate the expression of the NK gene complex (NKC) and Ly49F receptors after maturation, resulting in an enhanced lytic function. Consequently, in the mouse IL-21 is not essential for NK cell development, but it may influence their proliferation and their maturation into effectors cells.

In human, IL-21 was initially shown to stimulate the development of NK cells *in vitro *[37] and is involved in the acquisition of a mature KIR repertoire [38] from human bone marrow progenitors. IL-21 has also proven crucial to their adoption of a fully functional cytotoxic capacity [31,37]. On human mature NK cells, IL-21 was recently shown to downregulate NKG2D on NK and T cells stimulated by IL-15 [39].

Like in the mouse [18], our results suggest that IL-2 and IL-15 induce similar effects in the human NK cells, both cytokines being able to induce the expression of KIR receptors in a fraction of KIR-negative populations. The addition of IL-21 prevents KIR expression, at least to some extent. The paucity of available KIR mAbs thus represents an obstacle to our study and prevents us from drawing the formal conclusion that the so-called KIR-negative NK cells do not express any activating or inhibitory KIRs. It remains unclear why IL-2 and IL-15, with or without IL-21, failed to modify significantly the KIR repertoire on the KIR-negative subpopulation analysed in NK bulk populations (see Figure 2 and Table 1). The mechanisms underlying the repertoire of a given NK population are not well defined. NK cells do not express all their germline-encoded receptors; instead, a selected combination of these receptors is expressed in a stochastic manner [40]. In the mouse, the pattern of expression of Ly49 receptors is determined by probabilistic transcriptional switches in the promoter regions of the genes and a similar mechanism may exist for KIR [41]. Cytokine environment and infection shape the NK repertoire, which reflects a balance between activating and inhibitory receptors. Signals delivered by cytokines through the JAK/STAT (Janus kiase/signal transducer and activator of transcription) transduction pathway modulate the expression of the different receptors on NK cells to preserve an equilibrium, maintaining self-tolerance and the capacity to produce cytokines or to be cytotoxic. By placing a KIR-negative population in culture, this equilibrium may be significantly modified, resulting in the reactivation or resetting of KIR genes that in turn are susceptible to cytokines.

We confirm recent data reported by Burgess and coworkers [24]; those investigators reported that IL-21 inhibited DAP10 expression, leading to the downregulation of NKG2D [24]. In addition, we show that IL-21 can inhibit expression of DAP12 (Figure 5). The fact that DAP12 also mediates NKp44 transduction signalling but not that of NKp40 and NKp46 [21] may account for the downregulation of NKp44 by IL-21. Inhibition of DAP12 has several controversial effects. In the mouse DAP12 knockout model, NK cells developed normally but the activating Ly49 receptors were downregulated and nonfunctional [42]. However, recent data also suggest that the absence of DAP12 correlates with potent tumour rejection mediated by NK cell activation [25]. Our results corroborate these data [25], showing that the NKp44-negative population, which has downregulated DAP12, is more cytotoxic than the NKp44-positive population. Because DAP12 is also required for the expression and signalling of KIR, mainly the KIR activator, we speculate that the reduction of KIR expression observed after 7 days of culture with IL-21 shown in Figure 3b,c (right columns) could be due, at least in part, to the downregulation of DAP12.

In summary, our results strongly suggest that it is possible to modify and ultimately even shape the repertoire of NK cells receptors by modulating *ex vivo *mature NK cells by means of cytokines. The addition of IL-21 to IL-15 or IL-2 increases significantly the number of cells, with unaltered capacity to produce IFN-γ and even more potent cytotoxic activity, which is quite similar to the effect of IL-21 on murine NK cell biology [18,41]. With regard to immunotherapy, this insight is fundamental because with this *ex vivo *strategy it may become possible to envisage tailoring specific phenotypes to subpopulations of NK cell. Human cancer patients would certainly be the first to benefit from a strategy consisting of NK cell based immunotherapy, but one may also envisage, in the near future, treatment of autoimmune diseases after amplifying *ex vivo *NK cell populations possessing regulatory properties [43].

## Abbreviations

CFSE = 5-carboxyfluorescein diacetate succinimidyl ester; DAP-12 = DNAX-activating protein of 12 kDa; FACS = fluorescence-activated cell sorting; FCS = foetal calf serum; IFN = interferon; IL = interleukin; KIR = killer cell immunoglobulin-like receptor; mAb = monoclonal antibody; NCR = natural cytotoxicity receptor; NK = natural killer; NKG2D = NK group 2D; PBMC = peripheral blood mononuclear cell; PBS = phosphate-buffered saline; PCR = polymerase chain reaction; rh = human recombinant.

## Competing interests

The authors declare that they have no competing interests.

## Authors' contributions

CdR designed, performed, analyzed and interpreted experiments and wrote the paper. SFL designed, performed, analyzed and interpreted experiments, contributed to protocol design and wrote the paper. SJ and GS performed experiments. JMD was responsible for intellectual and financial contributions. JV designed and interpreted experiments, wrote the paper, designed the protocol, and oversaw all aspects of the experiments.
